# Superior Antioxidant Capacity of *Berberis iliensis*—HPLC-Q-TOF-MS Based Phytochemical Studies and Spectrophotometric Determinations

**DOI:** 10.3390/antiox9060504

**Published:** 2020-06-09

**Authors:** Saniia Abdykerimova, Zuriyadda Sakipova, Sylwia Nakonieczna, Wojciech Koch, Anna Biernasiuk, Aneta Grabarska, Anna Malm, Kaldanay Kozhanova, Wirginia Kukula-Koch

**Affiliations:** 1 School of Pharmacy, S.D. Asfendiyarov Kazakh National Medical University, Tole-bi 94, 050012 Almaty, Kazakhstan; abdykerimova.s@kaznmu.kz (S.A.); sakipova.z@kaznmu.kz (Z.S.); kozhanova.k@kaznmu.kz (K.K.); 2 Chair and Department of Pharmacognosy, Medical University of Lublin; 1, Chodzki str., 20-093 Lublin, Poland; sylwia.nakonieczna2@gmail.com; 3 Chair and Department of Food and Nutrition, Medical University of Lublin; 4a, Chodźki str., 20-093 Lublin, Poland; kochw@interia.pl; 4 Department of Pharmaceutical Microbiology with Laboratory for Microbiological Diagnostics, Medical University of Lublin, 1, Chodźki str., 20-093 Lublin, Poland; anna.biernasiuk@umlub.pl (A.B.); anna.malm@umlub.pl (A.M.); 5 Department of Biochemistry and Molecular Biology, Medical University of Lublin, 1, Chodźki str., 20-093 Lublin, Poland; aneta.grabarska@umlub.pl

**Keywords:** berberidaceae, barberry, HPLC-Q-TOF-MS, antimicrobial activity, antioxidant potential, natural products, isoquinoline alkaloids, polyphenols, Kazakh flora

## Abstract

The aim of the present study was to determine the composition, antiradical and antimicrobial activity of fruits, leaves and roots of an underestimated species of barberry—*Berberis iliensis*—growing in Kazakhstan. Particular attention was paid to the determination of the composition of its extracts by high-performance liquid chromatography coupled with mass spectrometry (HPLC-ESI-Q-TOF-MS) analysis. As a result of the chromatographic and spectrometric study 33 secondary metabolites from the groups of phenolic acids and their esters, flavonoids, alkaloids and organic acids were identified and 15 of them—quantified. The isomers of caffeoyl-glucaric acid, caffeic acid derivatives, isoquercetin, berberine and jatrorrhizine were the most abundant components of the tested extracts. The antiradical activity tests were performed by 2,2-diphenyl-1-picrylhydrazyl (DPPH) and Folin-Ciocalteu assays on four types of extracts (water, ethanol, ethanol-water 7:3 *v/v*, ethanol-water 1:1 *v/v*) from the three organs of the plant. The highest antiradical potential (IC50 = 80 ± 6.36 µg/mL) and phenolic content (440 ± 17.1 mg gallic acid equivalents/L) was calculated for ethanol- water (1:1 *v/v*) extracts from the leaves and could be influenced by the abundant presence of simple phenolic acids, flavonoids and glucaric acid esters. Among reference microorganisms, *M. luteus*, *S. epidermidis*, some *S. aureus* and *B. cereus* belonging to Gram-positive bacteria and yeasts from Candida species were the most sensitive to roots extract that was found the most active among the studied samples. The results of the study classify *Berberis iliensis* as a strong antioxidant agent and as a plant with an antimicrobial potential.

## 1. Introduction

*Berberis* L. is the most widely represented genus of the Berberidaceae botanical family. It consists of more than 500 species of shrubs that are growing naturally and as ornamental plants around the world in most of climate conditions except from humid or particularly hot areas [1]. Barberry species are edible plants. For centuries the fruit, leaves, bark and roots of *Berberis* shrubs were considered an ailment for various diseases [2], including internal bleeding (China, England) or liver and gall bladder diseases (USA). A decoction from barberry berries was known already in the Antiquity when “yellow fever” raged accompanied by a “rotten diarrhea.” At those times, barberry berries were referred to as “purifying the blood” [2]. Currently *Berberis* species are widely used around the world. In Central Asia, where this species occurs commonly, its medicinal forms are administered as anti-scurvy, choleretic, antioxidant, astringent, hemostatic, antipyretic and antimicrobial agents, as well as for catarrhal diseases and conjunctivitis [3].

Traditional pharmacological applications of barberry species were based on the content of alkaloids that influenced the physiological impact of the shrubs. The root, twigs and the root bark were perceived as the major sources of alkaloids from different subgroups of isoquinolines, including protoberberines, aporphines, benzylisoquinolines and bisbenzylisoquinolines. Many of these bright yellow or orange components are present in the form of organic cations with four linked benzene rings and one nitrogen atom that is binding the two pairs of rings. A wide variety of compounds have been isolated from different representatives of *Berberis spp.*; however, berberine, berbamine, palmatine, oxyacanthine and jatrorrhizine were the highest represented in all studied plants [1,4].

In the folk medicine of Tajikistan and Uzbekistan, an infusion of barberry roots is still used for neurasthenia, rheumatism, fever, cardiovascular diseases and external inflammatory processes. The root bark is a dye that colors wool and silk in golden, bright yellow and brown shades. No less important is the nutritional use of fruits both in fresh form and as a seasoning for meat dishes, for making jam or pastilles [5]. The herein studied species, *Berberis iliensis* M.Pop. that is widely spread in Kazakhstan is drunk for headaches, nosebleeds, jaundice, stomach diseases and its aboveground parts are popularly used as a hemostatic, anti-inflammatory and antimicrobial agent. Decoctions and infusions from barberry well quench thirst in feverish patients, excite appetite, have a mild laxative effect, increase the secretion of gastric juice and improve digestion [6]. According to Pozharskiy and Chekalin [7], up to 9 barberry species were described to grow wild in Kazakhstan, including *B. iliensis* that is a rare endemic plant growing in this region.

The multitude of traditional uses of *B. iliensis* encouraged the authors to take a closer look at the composition and its potential influence on the biological activity of this underestimated barberry species that *B. iliensis* is. The aim of this study was to determine the qualitative and quantitative profiles of different organs of the plant using the HPLC-DAD-ESI-Q-TOF-MS instrumentation (high-performance chromatograph coupled with electrospray ionization mass spectrometer), to quantify the major compounds in its extracts and to focus on the determination of the antioxidant and antimicrobial properties of the samples. So far this species has been studied only for the alkaloids’ content. This work aims at the determination of alkaloids’ but also phenolics’ profile of the plant as the advantages of free radicals scavengers’ intake are of high concern these days.

## 2. Materials and Methods

### 2.1. Chemicals and Reagents

Reagents used to perform the antioxidant determinations—2,2-diphenyl-1-picrylhydrazyl (DPPH) and Folin-Ciocalteu reagent, the standards of gallic acid, chlorogenic acid, berberine, palmatine, jatrorrhizine, magnoflorine, quercetin, D-saccharic acid (glucaric acid), glucaric acid potassium salt and rutoside at purity exceeding 95% were purchased from Sigma-Aldrich (St. Louis, MO, USA). Spectroscopic grade solvents used for the LC-MS analyses—acetonitrile, water and formic acid, were manufactured by Merck (Darmstadt, Germany). The reagent grade chemicals used for the preparation of extracts and spectrophotometric determinations were purchased from Avantor Performance Materials (Gliwice, Poland).

### 2.2. Plant Material

Plant material investigated in the present study represented the roots, leaves and fruits of *Berberis iliensis* M.Pop. They were collected in the Charyn canyon of the Almaty region (Kazakhstan) at the beginning of flowering period (leaves) and late autumn (fruits and roots). The collected species was authenticated by the director of the Institute of Botany and Phytointroduction, Almaty, Kazakhstan, doctor G. Sitpayeva. Dried plant material used in the study is kept in the Department of Pharmacognosy at the Medical University of Lublin, Poland, by the corresponding author under the voucher specimen numbers WKK18007 to WKK18009.

### 2.3. Extraction

Initially all plant parts were ground and powdered using an electric mill (type WZ-1, ZBPP, Poland). Next, 1 g of each tested organ—roots, leaves and fruits was extracted with 10 mL of the extracting solvent—ethanol, ethanol-water (1:1 *v/v*), ethanol-water (7:3 *v/v*) or pure deionized water. The extraction was performed three times, 30 min each, at room temperature using ultrasonic bath. Later the extracts were filtrated and centrifuged 10 min long at 3500 rpm. The supernatant was collected, placed in weighted 5 mL Eppendorf tubes and evaporated to dryness at 45 °C using an Eppendorf Concentrator Plus (Hamburg, Germany). The obtained sample were refrigerated at 4 °C until further investigations that took place within one-week time.

### 2.4. The Conditions of Qualitative and Quantitative HPLC-ESI-Q-TOF-MS Analyses

The chromatographic separation of the extracts, the identification of secondary metabolites and the quantification of the major constituents were performed using an analytical platform HPLC-ESI-Q-TOF-MS 6500 series by Agilent Technologies (Santa Clara, CA, USA) that was composed of an HPLC chromatograph (1200 Series) built from an autosampler (G1329B), binary pump (G1312C), photodiode array detector—DAD (G1315D) and degasser (G1322A) and the Q-TOF-MS mass spectrometer (G6530B). The analytes were separated using Zorbax Eclipse Plus RP-18 chromatographic column (dimensions: 150 mm × 2.1 mm; dp = 3.5 μm) also by Agilent Technologies (Santa Clara, CA, USA). The following operating conditions were applied in the HPLC chromatograph—the flow rate 0.2 mL/min, the thermostat temperature 25 °C, the injection volume 20 μL, the DAD detection at 254, 280, 320 and 365 nm in the range of 190–600 nm, the run time 45 min. The mobile phase contained 0.1% formic acid in water (solvent A) and 0.1% formic acid in acetonitrile (solvent B). The composed gradient elution: 0 min: 98% of A; 5 min: 90% of A; 20 min: 60% of A; 35 min: 5% of A; and 36 min: 98% of A [8].

Mass spectra were recorded in negative and positive ionization settings within the *m/z* range of 100–1200 Da, with the capillary voltage of 3.5 kV, gas flows of 12 L/min, gas and sheath gas temperatures of 350 and 325 °C, respectively, fragmentation voltage of 150 V, skimmer voltage of 65 V and the collision energies of 10 and 20 V. In the method the tandem MS/MS spectra were acquired for the two most intense peaks in every scan, however, after the collection of one MS/MS spectrum of a given compound this m/z signal was excluded for the following 0.2 min. In this way fewer intensive signals could be also fragmented. The structure determination was based on the fragmentation spectra, solutions of standards, literature data, retention times and open databases (Metlin).

The quantitative determination of the selected components was based on a direct comparison with the injected standards. First the calibration curves of the standards were plotted based on the injections of 10 different concentrations in the range of 0.0005–0.05 mg/mL, in triplicate, obtained from the dilution of the stock solutions. The values of all regression coefficients (*R*^2^) exceeded 0.99. The quantification of selected metabolites was performed in the ranges much higher from the determined limit of detection (LOD) and limit of quantification (LOQ) values, in respect to the calculated linearity ranges for each reference compound. The method was formerly validated according to the methodology published by Kukula-Koch [8]. The quantification of secondary metabolites was performed by a direct comparison with reference compounds representing the same class of secondary metabolites. The content of flavonoid aglycones (quercetin and kaempferol) was calculated from the calibration curve of quercetin, flavonoids’ derivatives (quercetin glucuronide, isoquercetin, kaempferol glucoside)—from the calibration curve equation of rutoside, phenolic acids (chlorogenic acid derivatives)—from the calibration curve of chlorogenic acid, alkaloids (berberine, palmatine and magnoflorine)—from the same standard compounds, jatrorrhizine (alkaloid) from the calibration curve of palmatine and glucaric acid derivatives from the potassium salt of glucaric acid. Alkaloids were analyzed in the positive ionization mode, whereas the remaining compounds—in negative ionization mode.

### 2.5. The Determination of the Antioxidant Potential of the Obtained Extracts by Spectrophotometric Methods

#### 2.5.1. 2,2-Diphenyl-1-Picrylhydrazyl Assay

The extracts were tested to quench 2,2-diphenyl-1-picrylhydrazyl(DPPH) radical using a modified protocol described previously [9,10]. Briefly, several concentrations of each tested extract obtained by dilution in dimethyl sulfoxide—DMSO (range 50–10,000 µg/mL) were chosen to evaluate the IC_50_ value of the samples. In the protocol, 0.2 mL of each dilution of a sample was mixed with 1.8 mL of DPPH solution prepared immediately before determinations by diluting 6 mg of DPPH in 100 mL of ethanol. The solutions were mixed and incubated in the dark at room temperature for 30 min. Then, their absorbance was measured at a wavelength of 515 nm using a Unicam Helios Gamma (Thermo Electron Corporation) spectrophotometer. Water solution of gallic acid at a concentration of 0.1 mg/10 mL was used as a reference. The measurements were performed in triplicate for each extract.

#### 2.5.2. The Determination of the Total Phenolic Content (TPC)

The same extracts were tested in a Folin-Ciocalteu assay. This study is often perceived as the evaluation of the total phenolic content; however, some authors treat it also as a method to assess the reducing potential of the investigated samples. TPC determinations were performed using a modified protocol proposed by Singleton and co-investigators [11]. 0.25 mL of every stock solution (1 mg/mL) was diluted with 2.5 mL of a freshly prepared Folin-Ciocalteu reagent (17-fold dilution in water) in dark glass vials. Afterwards, 2 mL of 7.5% sodium carbonate was added and the mixture was left to stand in the dark for 30 min. After the incubation, the absorbance of the samples was measured at a wavelength of 765 nm, using water as a blank. Simultaneously, ten gallic acid solutions at a concentration range of 50–500 mg/L were prepared and subjected to the same protocol as the test solutions to receive the gallic acid calibration curve. Additionally, a blank solution was used to avoid background absorbance. After plotting the calibration curve the activity of the investigated extracts was read and expressed as gallic acid equivalents. All determinations were performed in triplicate and the obtained results were elaborated using Statistica 10.0 PL and MS Excel 2010 software.

### 2.6. In Vitro Anitimicrobial Assay

The examined barberry extracts were subjected to in vitro antibacterial and antifungal activity testing in the broth microdilution method described by the European Committee on Antimicrobial Susceptibility Testing (EUCAST) [12] and Clinical and Laboratory Standards Institute (CLSI) guidelines [13]. A panel of reference strains of microorganisms was used and included Gram-positive bacteria (*Bacillus cereus* ATCC 10876, *Bacillus subtilis* ATCC 6633, *Micrococcus luteus* ATCC 10240, *Staphylococcus aureus* ATCC 6538, *Staphylococcus aureus* ATCC 25923, *Staphylococcus aureus* ATCC 43300, *Staphylococcus epidermidis* ATCC 12228), Gram-negative bacteria (*Bordetella bronchiseptica* ATCC 4617, *Escherichia coli* ATCC 25922, *Klebsiella pneumoniae* ATCC 13883, *Pseudomonas aeruginosa* ATCC 9027 and *Salmonella typhimurium* ATCC 14028) and fungi belonging to yeasts (*Candida albicans* ATCC 10231, *Candida glabrata* ATCC 90030, *Candida parapsilosis* ATCC 22019 and *Candida krusei* ATCC 14243) (see also Figure S1 in the Supplementary Material file).

The studied bacteria and fungi were purchased from American Type Culture Collection (ATCC), routinely used for the evaluation of antimicrobial properties. All the used microorganisms were first subcultured on the Sabouraud agar or suitable nutrient agar for 18–24 h at 35 °C or for 24–48 h 30 °C for bacteria and fungi, respectively. Samples containing studied *Berberis iliensis* extracts were dissolved in dimethyl sulfoxide (DMSO). Ciprofloxacin or nystatin (Sigma Aldrich, St. Louis, MO, USA) were used as a standard antibacterial or antifungal compounds, respectively.

The MIC (Minimal Inhibitory Concentration) of these extracts was examined by the microdilution broth method, using their two-fold dilutions in MH (Mueller-Hinton) broth (for bacteria) and RPMI 1640 broth with MOPS (Roswell Park Memorial Institute 1640 broth buffered 3-(N-morpholino) propanesulfonic acid) (for fungi) prepared in 96-well polystyrene plates. Final concentrations of extracts ranged from 20 mg/mL to 0.01 mg/mL. The bacterial and fungal suspensions were prepared in ampoules with physiological NaCl. Their optical density was 0.5 according to the McFarland standard. Next each microbial suspension was added per each well containing broth and various concentrations of the studied extracts. After incubation (in conditions as above) the MIC (Minimal Inhibitory Concentration) was determined based on the spectrophotometric assay where the lowest sample concentration showing complete microbial growth inhibition was noted down. Additionally growth, DMSO and sterile controls were carried out. The media with no barberry extracts were used as controls.

The MBC (Minimal Bactericidal Concentration) or MFC (Minimal Fungicidal Concentration) are the lowest concentrations of the tested samples that is required to kill a particular bacterial or fungal species. MBC and MFC were determined by removing the cultures used for MIC determinations from each well and transferring them on the appropriate freshly prepared agar medium (for bacteria and fungi). Next, the plates were incubated in conditions as above. The lowest concentrations of the tested samples with no visible growth observed were assigned as bactericidal or fungicidal concentrations. In this study, MBC/MIC and MFC/MIC ratios were also calculated for the determination of bactericidal/fungicidal (MBC/MIC ≤ 4, MFC/MIC ≤ 4) or bacteriostatic/fungistatic (MBC/MIC > 4, MFC/MIC > 4) effects of the studied extracts [14,15].

## 3. Results and Discussion

### 3.1. The Extracts Profiling by HPLC-ESI-Q-TOF-MS

*Berberis iliensis* has been an underestimated and insufficiently investigated plant species. For centuries it has been perceived as an alkaloid-containing plant, whereas the outcomes of current research clearly show a plentitude of secondary metabolites of different kind that are present mainly in its leaves and fruits. Polyphenols are certainly a well-represented group in its extracts. According to the Authors’ knowledge, this work shows the composition of phenolic compounds in the extracts of *B. iliensis* organs for the first time.

**Figure 1:**
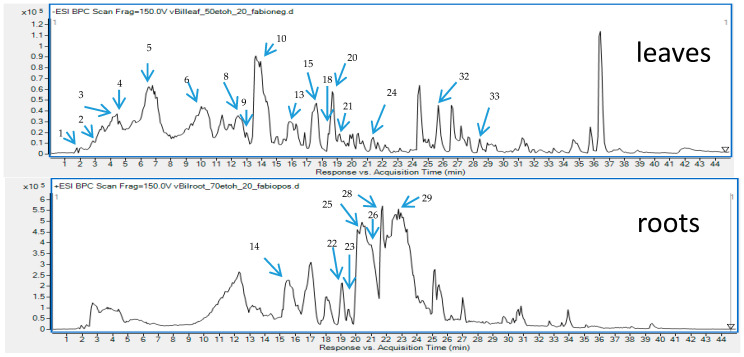
Total ion chromatograms recorded in the negative (**upper**) and positive (**lower**) ionization modes for the leaf and root extracts of *B. iliensis* with an assigned identification of major metabolites.

In total 33 different secondary metabolites were tentatively identified in various organs of the plant, based on the comparison with reference compounds, the analysis of scientific literature and the studies on their mechanism of fragmentation. Among the identified components that belong to the group of polyphenols—flavonoids (aglycones and glucosides), benzoic and cinnamic acids’ derivatives were found in the highest concentration. Also, simple organic acids and sugar acids from different organs of *B. iliensis* were present and are shown in Table 1 with the full list of identified metabolites in the tested samples. Next to simple phenolic acids like gallic acid, vanillic acid, chlorogenic acids, hydroxybenzoic acid, ferulic acid or caffeic acid, some esters and aldehydes of phenolic acids were also found, among them galloyl-glucose and its derivatives, coumaroyl-quinic acid, galloyl-glucose and others. Their chromatographic and spectrometric data were presented in the Table 1 and Figure 1 and they resembled the characteristics described in the scientific literature. Several papers underline their marked role in the antiradical activity, so their abundant presence in the extracts of leaves and fruits may certainly explain the biological functions of the plant [10,16].

Interesting derivatives of glucaric acid also called D-saccharic acid constituted the prevailing group of signals in the leaf extracts of *B. iliensis*. Beside the leaves, the four identified derivatives were also present in the fruits of barberry but at a much lower concentration (see Section 3.2. and the structures in the Figure S2 in the Supplementary Material file).

From chemical point of view, these compounds are esters formed between one to four acylated residues and glucaric acids. These derivatives have been thoroughly analyzed by some authors that mentioned their presence in some plant species like *Leonurus spp.* [19], *Citrus spp*. [37], cruciferous vegetables, grapefruits, oranges and apples [38]. Surprisingly, only one study on the composition of barberry extracts mentions their presence in the fruits of *Berberis microphylla* in large quantity, however, with no proposition on the type of caffeoyl ester substitution [39]. The authors have found no information on their presence in the leaves of barberry shrubs.

Only a few references focus on the biological value of glucaric acid derivatives. Among them, Żółtaszek and colleagues [38] mention several physiologically important features of D-glucarates, that include their antiproliferative, anti-inflammatory, immunostimulating and antioxidant properties.

The identification of the chemical type of caffeoyl-glucaric acid connections was possible thanks to the observations of Garran and colleagues [19] who, using a similar methodology (UHPLC-Q-TOF-MS), performed a systematic study on the identification of glucaric acid isomers. Based on these data, four caffeoyl-glucaric acid derivatives were identified in the leaves and fruits of *B. iliensis*, namely, 3-, 4-, 2- and 5-caffeoylglucaric acids in this particular elution order. All of them had the *trans* conformation. In their work, the authors described a characteristic fragmentation of the compounds. In our chromatograms and MS/MS spectra, we observed a similar behavior. The total ion chromatograms (TIC) of caffeoyl-glucaric acid derivatives provided a strong signal at 371 *m/z* in the negative ionization mode [M-H]^−^ that was fragmented to the following product ions: 209 *m/z* for [glucaric acid-H]^−^ and 191 *m/z* for [glucaric acid-H_2_O-H]^−^ (Table 1).

According to the recorded data, fruits of the studied barberry species were found to possess the highest diversity of phenolic compounds, whereas the leaves—the highest quantity. This information is particularly important as they both constitute a renewable plant material, that may be collected with no harm to the plant that becomes to be an endangered one [7] in Kazakhstan, with a decreasing area of distribution.

In comparison to the previously studied fruits of *Berberis microphylla*, *Berberis iliensis* fruits contain a smaller quantity of double-substituted quinic acid derivatives. The LC-MS analysis did not reveal the presence of any dicaffeoylquinic acid derivatives or dicaffeoylglucaric acid derivatives, that are highly represented in *B. microphylla* fruits. Also, the Kazakh species was richer in caffeic acid derivatives or esters and did not contain ferulic acid derivatives that were described by Ruiz and collaborators [39].

In comparison to the previously published research data on the composition of barberry species, similar metabolites that are reported in the Table 1 can be found in other species of barberry shrubs. However, the majority of publications on phenolic content of barberries mostly focus on the composition of their fruits that have been widely used in cuisine as non-toxic digestive agents in the production of juices, wines and jams or the roots [39]. Here, the obtained data pay attention to the leaves of the plant as they occurred to deliver the highest quantity of polyphenols in this particular species. In the former study on *Berberis sibirica*, similar phenolic components were identified in the overground parts of the plant [10]. Among phenolics, in this particular Siberian species the following phenolic acids were identified—*p*-coumaric, caffeic, syringic, gallic, chlorogenic, protocatechuic, sinapinic acids and flavonoids—luteolin and apigenin.

The chromatograms obtained for the root samples of *B. iliensis* revealed the presence of some simple phenolic compounds. The roots contained quinic acid, chlorogenic acid, hydroxybenzoic acid derivatives and small quantities of quercetin, hesperetin and rosmarinic acid. However, the composition of the roots was dominated by high content and diversity of alkaloids that belong to the group of isoquinolines. Majority of the identified alkaloids were also found in the leaves and even in the fruits but in noticeably smaller concentrations. In this study, the authors confirmed the findings of other research groups that focused on the determination of alkaloids’ composition in this species [27]. Similarly, to the other results, berberine, palmatine, jatrorrhizine, magnoflorine, berbamine, N-methylcoclaurine, obaberine, berbamunine and oxyacanthine were identified in the investigated samples. However, based on the LC-MS spectra, the authors could not distinguish berbamunine from obaberine (Table 1).

### 3.2. Quantitative Analysis of Alkaloids and Phenolics in the Extracts

**Figure 2:**
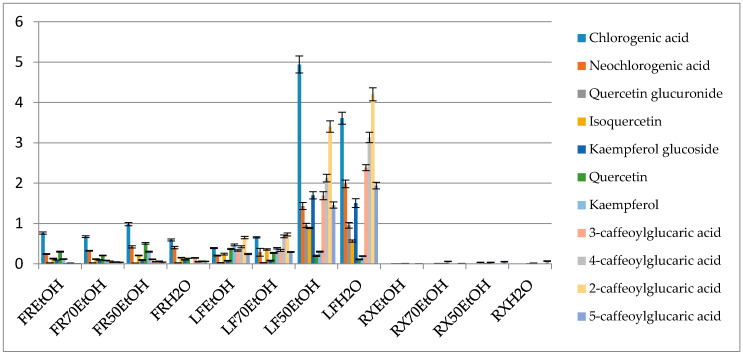
Percentage content of the selected metabolites in the obtained extracts (FR—fruit, LF—leaf, RX—root, EtOH—96% ethanol extract, 70EtOH—70% ethanol extract, 50EtOH—ethanol-water 1:1 (*v/v*) extract, H_2_O—water extract).

The conducted studies have shown that the composition of *B. iliensis* extracts depends on the solvent used and the organ analyzed (Figure 2 and Figure S2 in the Supplementary Material file). Ethanol-water (1:1 *v/v*) and water were the favorable extracting solvents in terms of the extraction of phenolic compounds. Leaves were found to contain the highest quantity of polyphenols and stayed in contrast with the roots that contained only several phenolic components and at a low concentration.

The profile of phenolic compounds that was obtained in the mass spectrometry-based analysis showed the prevalence of caffeic acid derivatives. This compound was present in a free form and built the chlorogenic acid derivatives or the esters of glucaric acid. Chlorogenic acid was the most abundant metabolite in the extracts and in the ethanol-water (1:1 *v/v*) leaf extract it had a concentration of 4.94 ± 0.21%. The following compounds were also quantified in the same leaf extract—neochlorogenic acid (1.43 ± 0.086%), kaempferol glucoside (1.69 ± 0.089%), quercetin glucuronide (0.95 ± 0.051%) and caffeic acid esters of glucaric acids. The latter were most abundant in the water extract from the leaves and provided 4.20 ± 0.16, 2.38 ± 0.074, 3.13 ± 0.13, 1.93 ± 0.082% for 2-, 3-, 4- and 5-caffeoylglucaric acids, respectively.

Similarly, to the study on *Beberis microphylla*, it could be concluded that summed glucaric acid derivatives’ contents constituted a similar number to other summed phenolics [39].

**Figure 3:**
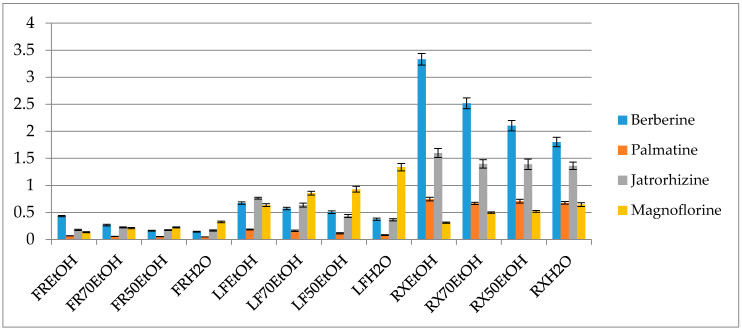
Percentage content of the selected alkaloids in the extracts (FR—fruit, LF—leaf, RX—root, EtOH—96% ethanol extract, 70EtOH—ethanol-water (7:3 *v/v*) extract, 50EtOH—ethanol-water (1:1 *v/v*) extract, H_2_O—water extract).

Interestingly the variation in the concentration ratio between alkaloids occurs in various organs of the plant (Figure 3). The leaves are better sources of magnoflorine (1.33 ± 0.069% of the ethanol extract), whereas they contain smaller quantities of protoberberines like berberine, palmatine or jatrorrhizine in comparison to the roots (Figure 3 and Tables S1 and S2 in the Supplementary File). On the other hand, root extracts contain berberine and jatrorrhizine in the highest concentrations, however, their composition is very rich also in the derivatives of other subgroups of alkaloids. According to the study, ethanol extracts from the roots contained the highest quantity of isoquinolines with 3.33 ± 0.1% of berberine, 1.59 ± 0.083 % of jatrorrhizine, 0.74 ± 0.03% of palmatine and 0.31 ± 0.011% of magnoflorine. Fruits were characterized by the smallest content of alkaloids but the diversity of compounds was similar to that of the leaves.

A thorough HPLC-QQQ-MS based quantification of alkaloids from eight different barberry species was published by Singh and co-investigators [40]. In their work, roots were also found to contain the highest concentration of alkaloids. Among the studied compounds, berberine, jatrorrhizine, palmatine and magnoflorine were in the highest concentration in *B. lycium* (262.3 mg/g), in *B. lyceum* (32.4 mg/g), *B. aristata* (89.2 mg/g) and *B. petiolaris* (285 mg/g), respectively. Based on these results and also other numerous publications on the quantity assessment of alkaloids in barberry species our results stay slightly higher above the average content of alkaloids in *Berberis spp.* No quantification of alkaloids in *B. iliensis* have been performed so far.

It is worth a note that the concentration of alkaloids in the plant organs may vary depending on the age of the plant. Srivastava and colleagues mention a mean value of berberine content in differently aged organs of the Indian barberry species. In their work, they showed that the mean concentration of berberine in the young shoots was calculated as 0.04%, whereas in the young perynchymatous roots as 1.41% [4].

### 3.3. The Determination of the Antioxidant Potential of Various Organs and Extracts from Berberis iliensis

The antioxidant activity assessment shows both a marked influence of extracting solvent on the content of antiradical compounds in the extracts and noticeable differences between the plant’s organs (Table 2).

From all tested organs of *Berberis iliensis*, leaves were characterized by the highest antioxidant activity which was in agreement with chromatographic determinations that also proved that this part of the plant is the richest in phenolics.

Taking into account the data presented in Table 1 and Figure 1, it could be noticed that the composition of root extracts is based on alkaloids. Roots contained only trace amounts of phenolics and therefore its antioxidant activity was very weak. On the other hand, fruit extracts from *B. iliensis* were of moderate antiradical activity, as they contained medium amounts of polyphenols, of which chlorogenic acid was determined in the highest amounts. The influence of the solvent system on chemical composition and antioxidant activity was ambiguous. In the case of the leaves, extracts obtained using a mixture of ethanol-water (1:1 *v/v*) and water were considered the richest in phenolics and characterized by the highest antioxidant activity. However, regarding fruits and roots, water solutions of ethanol (both concentrations) were considered the most efficient towards extraction of various polyphenols from these two parts of *B. iliensis*.

A higher antiradical activity of more polar extracts may also be influenced by the presence of glucaric acid esters that are more efficiently extracted with high polarity solvents (water or ethanol-water 1:1 *v/v*). These compounds seem to have an important impact on the antiradical and antiproliferative capacity of the plant material, vegetables and fruits [38]. In this study, their highest content was determined in the leaves of *B. iliensis*, whereas root extracts did not reveal their presence. It is worth noting that in the herein described leaf extracts, the total content of the four identified caffeoyl esters of glucaric acid was equal to the sum of all other major phenolics, so their impact on the total activity may be meaningful.

The results of DPPH test and the total phenolics determinations using F-C reagent were in a good agreement, what was to be expected, as the latter one is very often described as one of the antioxidant parameters [41].

Various species of barberry, especially their fruits, are known for its high antiradical potential. Their constituents, including simple phenolic acids and flavonoids, have been described in the scientific literature as strong antiradical agents [10,42,43].

Özgen and co-investigators [44] determined the antioxidant activity of *Berberis vulgaris* fruits using several in vitro tests—trolox-equivalent antioxidant capacity (TEAC), ferric ion reducing antioxidant power (FRAP) and total phenolic content (TPC). The latter parameter was determined to be 2962 mg of GAE per 1 L of fruit juice. This value is hard to compare with the results of the present study, however if we take into account that in this study we used the extracts at the concentration of just 1 mg/mL, which was equal to 1 g of fresh organs, the antioxidant activity of fruits and especially leaves, of *Berberis iliensis* seems to be much higher in comparison to the fruits of *Berberis vulgaris*. Previous studies of Kukula-Koch and co-workers [9] revealed that the most active extracts obtained from Cretan barberry herb (*Berberis cretica* L.), a plant which is widely known for its high antioxidant activity, were characterized by similar antiradical potential in DPPH and F-C assays. Moreover, the most efficient extraction method was also a mixture of ethanol-water (1:1 *v/v*), which is in agreement with the present study.

It should also be emphasized that the antioxidant potential of *B. iliensis* (especially leaves) is high in comparison to other natural products which are widely known for its significant antioxidant activity—tea and various species of berries. TPC of various black and green teas was established in a range of 175–582 mg/L for black teas and 636–973 mg/L for green teas, which is on a similar level for the most active extracts evaluated within the present research [45,46]. The leaves of *B. iliensis* were characterized by so high antioxidant activity that may be compared even with a plant which is one of the richest in polyphenols—different species of berries. Zapata and co-workers [47] who investigated *Vaccinium meridionale*—a berry known for its high polyphenols content and antioxidant activity, revealed that TPC parameter was up to 724 mg GAE/L, which is only twice higher than the results of the present study. Very high concentration of phenolics, confirmed in a F-C assay was also proved for other berries—maqui-berries (*Aristotelia chilensis*), bilberry and blackberry pomace extracts [48,49].

The constituents of the studied extracts contain metabolites that had been previously described as active radical scavengers. The study on the antioxidant potential of caffeic acid by Liu and collaborators [50] shows a distinct potential of this compound in relation to other hydroxyl- and methoxyl- substituted derivatives of benzoic and cinnamic acids. According to the results, caffeic acid is the second most active radicals’ scavenger from the tested ten phenolic acids and exhibits a 4 times stronger antioxidant efficacy in comparison to Trolox. Also, the study of Benbettaieb and colleagues describes the application of caffeic acid in hydrocolloid-based films due to its strong antioxidant potential [51]. Even if caffeic acid is not the major component of the studied extract, its role in the total scavenging potential of the barberry extracts may be meaningful.

Chlorogenic acid and kaempferol were found to be the major components that were responsible for the antiradical potential in the study of Kumari and collaborators on *Amaranthus viridis* [52]. The survey of Chen and colleagues [53] suggests a similar antioxidant potential of chlorogenic acid to caffeic acid, so its role in the total antiradical potential may be significant.

Only a few publications describe the antioxidant potential of glucaric acid derivatives. Interestingly in combination with other radical scavengers, like resveratrol, glucaric acid even in low concentrations (0.1 or 0.5 mM) markedly reduced thrombin-induced platelet aggregation and the generation of superoxide radical. According to the authors these particular properties may be related to the synergistic action but its mechanism has not yet been fully determined [54].

Alkaloids are described as weak antioxidants in the studies of various authors. Many works underline strong anticancer or marked antimicrobial potential of alkaloids contrary to their antiradical potential. That is why some authors describe a stronger antiradical potential of the total extracts from barberries than single alkaloids [55,56]. According to Shirwaikar and colleagues [56] berberine, even if less potent, can reduce the DPPH radical to hydrazine in a reaction of hydrogen donors from its structure. Besides this molecule is able to remove oxygen, scavenge reactive nitrogen and oxygen species, bind metal ions and up-regulate the antioxidant defenses in humans.

Based on these reports it can be concluded, that the antioxidant potential of the tested samples is mainly influenced by the extracted polyphenols or organic acids.

### 3.4. The Antimicrobial Activity Assessment of Berberis iliensis Extracts

The antimicrobial assay indicated that the extracts from *Berberis iliensis* leaves, fruits and roots (ethanol-water 1:1 *v/v*) showed some potential activity with bactericidal or fungicidal effect (data presented in Table 3).

Generally, Gram-negative bacteria were more resistant to all studied extracts than Gram-positive microorganisms. It is possible that active compounds in these extracts can easily break important bonds in peptidoglycan of cell wall in Gram-positive bacteria. This structure in Gram-negative bacteria is far more complex and it is among other things why they are more resistant for biologically active substances. The other fact is that phenolic compounds or alkaloids, which are present in studied extracts, generally show antimicrobial activity against Gram-positive bacteria [57].

The obtained results showed that among the studied barberry extracts, roots extract was the most active antimicrobial with a higher effect against reference *Micrococcus luteus* ATCC 10240 and *Staphylococcus epidermidis* ATCC 12228 strains. The lowest inhibitory and bactericidal concentrations were 0.02–0.31 mg/mL and 0.02–0.62 mg/mL against these bacteria, respectively. Other Gram-positive microorganisms were also highly sensitive to this extract, *viz Staphylococcus aureus* ATCC 25923, *S. aureus* ATCC 6538 and *Bacillus cereus* ATCC 10876 (MIC = 1.25 mg/mL and MBC = 5 mg/mL). In the case of *S. aureus* ATCC 43300 and *Bacillus subtilis* ATCC 6633, antibacterial activity was similar (MIC = 2.5 mg/mL and MBC = 5 mg/mL).

The leaves extract had a higher effect towards reference *B. cereus* (MIC = 1.25 mg/mL, MBC = 2.5 mg/mL) and all strains of staphylococci *S. aureus* or *M. luteus* (MIC = 2.5 mg/mL and MBC = 2.5–>20 mg/mL). The activity of this extract against remaining bacteria *S. epidermidis* and *B. subtilis*, was slightly lower with MIC = 10 mg/mL and MBC ranging from 10 mg/mL to 20 mg/mL. In the case of *B. iliensis* fruits, the measured antimicrobial activity was slightly weaker. The most sensitive to this extract were *S. aureus* ATCC 6538, *S. aureus* ATCC 43300 and *M. luteus* from Gram-positive bacteria. The growth of remaining staphylococci and bacilli was inhibited at a concentration of 10 mg/mL and cells were killed at a concentration range of 10 mg/mL to >20 mg/mL.

Moreover, among Gram-negative rods-shaped, against *Bordetella bronchiseptica* ATCC 4617 the highest effect was shown by *B. iliensis* leaves extract (MIC = 2.5 mg/mL and MBC = 10 mg/mL) and slightly lower by other extracts (MIC = 5 mg/mL and MBC = 10 mg/mL). *Klebsiella pneumoniae* ATCC 13883 was sensitive to barberry fruits extract with the same MIC value (5 mg/mL) and MBC = 10–20 mg/mL). The susceptibility of the other Gram-negative bacteria was lower. The minimal concentrations of all extracts which inhibited growth or killed these microorganisms were 10–≥20 mg/mL.

Noteworthy is the particularly high activity of the *B. iliensis* roots extract towards reference fungi belonging to *Candida* species. The lowest inhibitory and fungicidal concentrations of this extract were 0.31–0.62 mg/mL and 2–8 mg/mL, respectively. This effect, similar to Gram-positive bacteria, may be associated with a high content of alkaloids, especially berberine. The activity of leaves and fruits extracts against yeasts was lower. The minimum concentrations of both extracts which inhibited growth of yeasts ranged from 5 mg/mL (*C. albicans* ATCC 10231, *C. parapsilosis* ATCC 22019 and *C. krusei* ATCC 14243) to 10 mg/mL (*C. glabrata* ATCC 90030). In turn, minimum concentrations which killed all fungi were 10 mg/mL.

Taking into account the MBC/MIC and MFC/MIC ratios, it was shown that generally extracts from *B. iliensis* leaves and fruits had bactericidal or fungicidal effect towards reference microorganisms (values of MBC/MIC or MFC/MIC ≤ 4). However, in the case of leaves extract, ratio MBC/MIC ≥ 8 indicated a bacteriostatic effect only against *S. aureus* ATCC 6538 and *S. aureus* ATCC 43300. In turn, for *Salmonella typhimurium* ATCC 14028, the coefficient of MBC/MIC for both extracts was not determined because MBC > 20. In the case of barberry roots extracts exhibited fungistatic effect (MFC/MIC = 8–16) towards reference *Candida* spp., except *C. parapsilosis* ATCC 22019, with fungicidal activity (MFC/MIC = 2).

As far as authors know, there are no previous reports about antimicrobial activity of extracts from *B. iliensis* leaves, fruits and roots, so it is not possible to further compare those results with other publications. However, there are a few data about antibacterial or antifungal effect of some extracts from other *Berberis* species.

According to Srivastava et al. [4], water, alcohol, ether and chloroform extracts from leaves, root and stems of different *Berberis* species (*B. vulgaris, B. chitria, B. heterophylla* and *B. aetnensis*) had some antimicrobial activity in vitro against Gram-positive or Gram-negative bacteria and fungi. In turn, other studies of the same authors [58] showed low antimicrobial activity of hydroalcoholic extracts of four *Berberis* species viz. *B. asiatica*, *B. aristata*, *B. lyceum* and *B. chitria*. *B. aristata* root extract exhibited low MICs values against *E. coli*, *B. cereus*, *S. aureus* and *Aspergillus flavus* and the stem extract—against *Streptococcus pneumoniae* and *B. cereus*. Moreover, the alcoholic and water extract of *B. chitria* roots were screened for their antimicrobial properties against microorganisms (*E. coli*, *S. aureus*, *B. proteus*, *S. typhii*) and the results showed that the alcoholic extract was found to be a better microbial agent than the water extract, which can prove a stronger effect of alkaloids that are better recovered from the plant matrix by alcohol [1].

Antibacterial or antifungal activity of extracts from *B. iliensis* leaves, fruits and roots may be associated with the presence of different active compounds (leaves and fruits—phenolic acids, for example, caffeic acid, chlorogenic acid or derivatives of glucaric acids, roots—alkaloids, for example, berberine, jatrorrhizine, palmatine and magnoflorine).

Phenolic acids are known to exhibit antimicrobial activity against a variety of microorganisms. Caffeic acid indicated a broad spectrum antimicrobial activity against reference pathogenic strains (*S. aureus*, methicillin-resistant *S. aureus*—MRSA, *E. coli*, *K. pneumoniae* and *C. albicans* with MIC ranging from 0.5 to 2.5 mg/mL, respectively [59]. Other authors showed antimicrobial effect of this acid against some bacteria (with MIC above ≥1024 μg/mL) [60] or towards *Clostridium botulinum* spores (at 0.78 and 3.25 mM, inhibited germination germination for 6 and 24 hr, respectively, with >100 mM required to render spores nonviable) [61]. Moreover, caffeic acid solutions exhibited bacteriostatic activity with MIC = 0.8 mg/mL against *Streptococcus mutans* [62]. Some data showed antifungal effect of caffeic acid against different *Candida* spp. (with MIC ≥ 1024 μg/mL or MICs and MFCs ≥ 4096 μg/mL) [60,63]. Several phenolic acids including caffeic and chlorogenic acid were active against strains of *L. monocytogenes* with bactericidal effect [64]. Moreover, chlorogenic acid may be used as a fungicide against phytopathogenic fungi, that showed the complete inhibition of spore germination or reduction of mycelial growth for *Sclerotinia sclerotiorum*, *Fusarium solani*, *Verticillium dahliae*, *Botrytis cinerea* and *Cercospora* sojina [65].

Alkaloids have also a broad spectrum of antimicrobial activity. According to some authors, berberine showed high antibacterial effect against *S. agalactiae* (MIC = 0.78 μg/mL) [66]. Other bacteria were also sensitive to berberine—*Shigella dysenteriae* (MIC = 25–300 μg/mL) [67], *S. aureus* and *E. coli* (MIC = 512 μg/mL) [68], *B. subtilis* (MIC > 512 μg/mL) [67], *Ps. aeruginosa* (MIC = 250–1000 μg/mL) [68,69,70]. Magnoflorine showed some antibacterial activity against *B. cereus* (MIC = 32 μg/mL, MBC = 128 μg/mL) and *S. aureus* (MIC = 64 µg/mL and MBC = 256 µg/mL), *E. coli*, *S. typhimurium*, *P. aeruginosa* (MIC = 512 μg/mL, MBC > 512 μg/mL) [71]. The MIC range of palmatine (500–1000 μg/mL) against *P. aeruginosa* was similar [70].

The sensitivity of yeasts is noteworthy. Alkaloids, especially berberine showed high effect against *Candida* species (*C. albicans −* MIC = 8–256 μg/mL [68,69], *C. tropicalis*, *C. parapsilosis* − MIC = 8 μg/mL, *C. krusei* − MIC = 4 μg/mL, *C. parapsilosis* − MIC = 16 μg/mL [69], *C. glabrata* − MIC = 256 μg/mL [68]) and *Cryptococcus neoformans* (MIC = 16 μg/mL) [69]. Magnoflorine had a similar effect against *C. albicans* (MIC = 50 μg/mL) [72]. The sensitivity studies carried out by Wang et al. [73] showed that the MICs of palmatine were in a range of 128–512 μg/mL against *C. albicans* strains and 64 ≥ 1024 μg/mL against non-*albicans Candida* (NAC) isolates (*C. krusei*, *C. parapsilosis*, *C. tropicalis*, *C. glabrata*, *C. guilliermondii*) [73]. Some data on palmatine [74] showed an antifungal activity with MICs ranging from 32 to 128 μg/mL against *C. albicans*, *C. tropicalis*, *C. parapsilosis*, *C. glabrata* and *C. krusei*. Moreover, according to other authors [75], jatrorrhizine was also effective against fungal species tested (MIC ranges from 62.5 to 125 µg/mL), while berberine and palmatine exhibited only marginal activity (MIC = 500 to ≥1000 µg/mL).

In accordance with the authors, a planar aromatic cationic center that is present in this type group of isoquinoline alkaloids is thought to be the primary pharmacophore responsible for both the antibacterial and antifungal activity and the recognition by efflux proteins in microbial cells, rendering them ineffective against efflux-resistant pathogens. Multiple bacteria and fungi along with selected protozoa and *Chlamydia* spp. are susceptible to these alkaloids in in vitro tests [1,76].

## 4. Conclusions

The herein described first comprehensive study on the Kazakh species of barberry shrub—*Berberis iliensis* shows a multitude of pharmacologically interesting secondary metabolites present in its extracts that belong to the groups of phenolics and alkaloids. The ethanol-water (1:1 *v/v*) extracts from the leaves and fruits and ethanol extracts from its roots were found to be the richest in secondary metabolites. According to the study, the leaves that favor the demand for renewable drugs’ sources exhibited the strongest antiradical properties and the most abundant composition in phenolic compounds like caffeic acid, benzoic acid and quercetin derivatives. Also, rare derivatives of glucaric acid were identified in the leaves. They may have a significant impact on the antiradical potential of the plant based on their high content in the leaf extract. On the other hand, the root extract was found to deliver the most promising results in terms of antimicrobial effects against Gram-positive microorganisms and yeasts due to its high alkaloids’ content. Therefore, it seems practical to use *Berberis iliensis* or its extracts in the future in the prevention and treatment of some civilization or infectious diseases.

## Figures and Tables

**Table 1:** The tentatively identified phenolics, organic acids and alkaloids in the extracts from *Berberis iliensis*.

No.	Ion.(+/−)	Rt.	Molecular Formula	*m/z* Calculated	*m/z* Experimental	Delta	RDB	MS/MS Fragments	Proposed Compound	Ref.	Fr.	Rx.	Fol.
**1**	−	2.69	C_6_H_8_O_7_	191.0197	191.0185	6.39	3	129, 111	**Citric acid**	[17]	+	+	−
**2**	−	3.1	C_13_H_16_O_10_	331.0671	331.0671	−0.09	6	241, 169, 125	**Galloyl-glucose**	[18]	+	−	−
**3**	−	4.2	C_15_H_16_O_11_	371.0620	371.0598	5.87	8	209, 129	**3-Caffeoylglucaric acid**	[19,20]	+	−	+
**4**	−	4.6	C_7_H_12_O_6_	191.0561	191.0568	−3.58	3.5	111	**Quinic acid**	[9,21]	−	+	+
**5**	−	6.6	C_15_H_16_O_11_	371.0620	371.0601	5.07	8	209	**4-Caffeoylglucaric acid**	[19]	+	−	+
**6**	−	9.8	C_15_H_16_O_11_	371.0620	371.0596	6.41	8	209, 129	**2-Caffeoylglucaric acid**	[19]	+	−	+
**7**	−	12.1	C_9_H_10_O_5_	197.0455	197.0459	−1.78	5	−	**Syringic acid**	[21]	+	−	−
**8**	−	12.5	C_15_H_16_O_11_	371.0620	371.0603	4.53	8	209, 112	**5-Caffeoylglucaric acid**	[19]	+	−	+
**9**	−	12.9	C_7_H_6_O_3_	137.0244	137.0255	−7.84	5	−	**Hydroxybenzoic acid isomers**	[22]	+	+	−
**10**	−	14.4	C_16_H_18_O_9_	353.0878	353.0897	−5.35	8	191, 129	**Chlorogenic acid**	[23]	+	+	+
**11**	−	14.7	C_8_H_8_O_3_	151.0401	151.0398	1.76	5	107	**Mandelic acid**	[24]	+	−	−
**12**	−	15.3	C_9_H_8_O_4_	179.035	179.0346	2.12	6	135, 120	**Caffeic acid**	[21]	+	−	−
**13**	−	15.4	C_16_H_18_O_8_	337.0929	337.0900	8.55	8	191, 173, 163	**Coumaroyl-quinic acid isomers**	[25]	+	−	+
**14**	+	15.6	C_20_H_24_O_4_N	342.1700	342.1680	−1.23	7	256, 192, 104	**Magnoflorine**	[26]	+	+	+
**15**	−	16.0	C_16_H_18_O_9_	353.0878	353.0895	−4.78	8	191, 129	**Neochlorogenic acid**	[23]	+	+	+
**16**	+	17.3	C_18_H_21_NO_3_	299.1521	299.1513	0.99	9.5	284, 252, 237	**N-methylcoclaurine**	[27]	+	+	+
**17**	+	17.8	C_37_H_40_N_2_O_6_	609.2959	609.2950	1.5	19	566, 381	**Berbamine**	[28]	tr	+	tr
**18**	+	18.0	C_37_H_40_N_2_O_6_	609.2959	609.2939	3.31	19	578, 566, 381	**Oxyacanthine**	[27]	+	+	+
**19**	−	18.4	C_27_H_30_O_16_	609.1461	609.1443	2.96	13	518, 300, 169	**Rutin**	[17]	+	−	−
**20**	−	18.7	C_16_H_18_O_9_	353.0878	353.0843	9.9	8	291,173	**(Z)-chlorogenic acid**	[23]	+	+	+
**21**	−	19.1	C_21_H_20_O_12_	463.0882	463.0843	8.4	12	300, 151	**Isoquercetin**	[29]	+	−	+
**22**	+	19.2	C_19_H_18_NO_4_	324.1230	324.1219	3.5	12	206, 121	**Dementhyleneberberine**	[30]	+	+	+
**23a,b**	+	19.3/19.6	C_38_H_42_N_2_O_6_	623.3116	623.3092	3.8	19	400, 268, 174	**Obaberine/berbamunine**	[27,31]	+	+	+
**24**	−	20.3	C_21_H_20_O_11_	447.0933	447.0897	8	12	301	**Quercetin glucoside**	[17]	+	−	+
**25**	+	20.5	C_20_H_20_O_4_N	338.1385	338.1379	2.33	12	126	**Jatrorrhizine**	[32]	+	+	+
**26**	+	21.6	C_19_H_15_NO_4_	322.1074	322.1075	−0.36	13	−	**Berberrubine**	[33]	+	+	+
**27**	−	21.9	C_9_H_10_O_4_	181.0506	181.0531	−13.6	5	108	**Syringaldehyde**	[34]	+	−	−
**28**	+	22.3	C_21_H_22_O_4_N	352.1543	352.1517	7.5	12	292, 155	**Palmatine**	[26]	+	+	+
**29**	+	22.8	C_18_H_22_NO_4_	336.1230	336.1214	4.88	13	231, 110	**Berberine**	[32]	+	+	+
**30**	−	24.4	C_20_H_18_O_11_	433.0776	433.0797	−4.76	12	300	**Guaiaverin**	[17]	−	+	−
**31**	−	24.5	C_16_H_14_O_6_	301.0718	301.0719	−0.46	10	240, 159	**Hesperetin**	[35]	−	+	−
**32**	−	24.6	C_15_H_10_O_7_	301.0354	301.0385	−10.34	11	229, 151	**Quercetin**	[17]	+	+	+
**33**	−	28.1	C_18_H_16_O_8_	359.0772	359.0762	2.89	11	228, 197	**Rosmarinic acid**	[36]	+	+	+

(Ion—type of ionization, Rt—retention time in minutes, delta—the error of measurement expressed in mmu, RDB—ring and double bond equivalent, Ref—references, Fr—fruit, Rx—root, Fol—leaf, tr—traces, + detected, − not detected; ‘23ab’—denotes to two peaks present in small retention time intervals that show the same m/z values. They are tentatively identified as obaberine or berbamunine, but the authors cannot indicate which peak stands for which compound).

**Table 2:** The antioxidant activity of the studied extracts.

Extract	DPPH [IC_50_ µg/mL]	F-C [mg GA/L]
**Fruits**		
Ethanol	1820 ± 150	32.4 ± 3.5
Ethanol-water (1:1 *v/v*)	620 ± 58.1	112 ± 10.1
Ethanol-water (7:3 *v/v*)	760 ± 55	55.6 ± 4.5
Water	1590 ± 128	38.7 ± 2.2
**Roots**		
Ethanol	4900 ± 342	21.1 ± 1.95
Ethanol-water (1:1 *v/v*)	3700 ± 250	26.5 ± 2.11
Ethanol-water (7:3 *v/v*)	3400 ± 230	24.3 ± 2.24
Water	5600 ± 420	17.6 ± 1.55
**Leaves**		
Ethanol	1590 ± 138	68.7 ± 5.52
Ethanol-water (1:1 *v/v*)	80 ± 6.36	440 ± 17.1
Ethanol-water (7:3 *v/v*)	604 ± 54.5	118 ± 10.9
Water	110 ± 10.5	372 ± 12.5
Gallic acid (0.01 mg/10 mL)	25 ± 1.02	NT

(NT—not tested).

**Table 3:** The activity data of *B. iliensis* leaves and fruits extracts expressed as Minimal Inhibitory Concentration (MIC), Minimal Bactericidal Concentration (MBC) or Minimal Fungicidal Concentration (MFC) [mg/mL] and MBC/MIC or MFC/MIC against the reference strains of bacteria and fungi.

Species of Microorganisms		MIC (MBC or MFC) [mg/mL] and {MBC/MIC or MFC/MIC} of the Studied Extracts and Controls (µg/mL)			
		Fruits (50:50)	Leaves (50:50)	Roots (50:50)	CIP or NY *
**Gram-positive** **bacteria**	*Staphylococcus aureus* ATCC 25923	10, (20), {2}	2.5, (10), {4}	1.25, (5), {4}	0.48, (0.48), {1}
	*Staphylococcus aureus* ATCC 6538	5, (20), {4}	2.5, (>20), {>8}	1.25, (5), {4}	0.24, (0.24), {1}
	*Staphylococcus aureus* ATCC 43300	5, (20), {4}	2.5, (20), {8}	2.5, (5), {2}	0.24, (0.24), {1}
	*Staphylococcus epidermidis* ATCC 12228	10, (20), {2}	10, (10), {1}	0.31, (0.62), {2}	0.12, (0.12), {1}
	*Micrococcus luteus* ATCC 10240	5, (10), {2}	2.5, (2.5), {1}	0.02, (0.02), {1}	0.98, (1.96), {2}
	*Bacillus subtilis* ATCC 6633	10, (10), {1}	20, (20), {1}	2.5, (2.5), {1}	0.03, (0.03), {1}
	*Bacillus cereus* ATCC 10876	10, (10), {1}	1.25, (2.5), {2}	1.25, (5), {4}	0.06, (0.12), {2}
**Gram-negative** **bacteria**	*Bordetella bronchiseptica* ATCC 4617	5, (10), {2}	2.5, (10), {4}	5, (10), {2}	0.98, (0.98), {1}
	*Klebsiellapneumoniae* ATCC 13883	5, (20), {4}	20, (20), {1}	20, (20), {1}	0.12, (0.12), {1}
	*Salmonella typhimurium* ATCC 14028	10, (>20), {>2}	20, (>20), {>1}	10, (20), {2}	0.06, (0.06), {1}
	*Escherichiacoli* ATCC 25922	10, (20), {2}	10, (20), {2}	10, (20), {2}	0.004, (0.004), {1}
	*Pseudomonas aeruginosa* ATCC 9027	10, (20), {2}	20, (20), {1}	10, (20), {2}	0.48, (0.98), {2}
**Fungi**	*Candida albicans* ATCC 10231	5, (10), {2}	5, (10), {2}	0.62, (5), {8}	0.48 *, (0.48), {1}
	*Candida parapsilosis* ATCC 22019	5, (10), {2}	5, (10), {2}	0.62, (1.25), {2}	0.24 *, (0.48), {2}
	*Candida glabrata* ATCC 90030	10, (20), {2}	10, (10), {1}	0.62, (10), {16}	0.24 *, (0.48), {2}
	*Candida krusei* ATCC 14243	5, (10), {2}	5, (10), {2}	0.31, (2.5), {8}	0.24 *, (0.24), {1}

The standard antibiotics used as positive controls (µg/mL): ciprofloxacin (CIP) for bacteria and nystatin (NY *) for fungi.

## Supplementary Materials

The following are available online at https://www.mdpi.com/2076-3921/9/6/504/s1, Figure S1: An example of the antimicrobial assay performed by the microdilution method (MIC) and agar plate assay (MBC), Figure S2: The structures of the metabolites present in the extracts, Table S1: Averaged percentage content of phenolic compounds in the studied extracts, Table S2: Averaged percentage content of the selected alkaloids in the obtained extracts.
