# Supplementary material for: Superior Antioxidant Capacity of Berberis iliensis—HPLC-Q-TOF-MS Based Phytochemical Studies and Spectrophotometric Determinations
Source: Antioxidants (Basel). 2020 Jun 9;9(6):504. doi: 10.3390/antiox9060504 (PMC7346193; doi:10.3390/antiox9060504)
Supplement: Supplementary file 1 [file antioxidants-09-00504-s001.pdf]

Superior Antioxidant Capacity of *Berberis iliensis*–HPLC-Q-TOF-MS Based Phytochemical Studies and Spectrophotometric Determinations

Saniia Abdykerimova [1], Zuriyadda Sakipova [1], Sylwia Nakonieczna [2], Wojciech Koch [3], Anna Biernasiuk [4], Aneta Grabarska [5], Anna Malm [4], Kaldanay Kozhanova [1], Wirginia Kukula-Koch [2,*]

[1] School of Pharmacy, S.D. Asfendiyarov Kazakh National Medical University, Tole-bi 94, 050012 Almaty, Kazakhstan; abdykerimova.s@kaznmu.kz (S.A.), sakipova.z@kaznmu.kz (Z.S.); kozhanova.k@kaznmu.kz (K.K.)

[2] Chair and Department of Pharmacognosy, Medical University of Lublin; 1, Chodzki str., 20-093 Lublin, Poland; sylwia.nakonieczna2@gmail.com (S.N.); virginia.kukula@gmail.com (W.K.-K.)

[3] Chair and Department of Food and Nutrition, Medical University of Lublin, 4a, Chodźki str., 20-093 Lublin, Poland; kochw@interia.pl (W.K.)

[4] Department of Pharmaceutical Microbiology with Laboratory for Microbiological Diagnostics, Medical University of Lublin, 1, Chodzki str., Lublin 20-093, Poland; anna.biernasiuk@umlub.pl (A.B.); anna.malm@umlub.pl (A.M.)

[5] Department of Biochemistry and Molecular Biology, Medical University of Lublin, 1, Chodzki str., 20-093 Lublin, Poland; aneta.grabarska@umlub.pl (A.G.)

* Correspondence: virginia.kukula@gmail.com; Tel.: +48814487087

Figure S1. An example of the antimicrobial assay performed by the microdilution method (MIC) and agar plate assay (MBC) (L – extract from leaves, F-extract from fruits of *Berberis iliensis*)

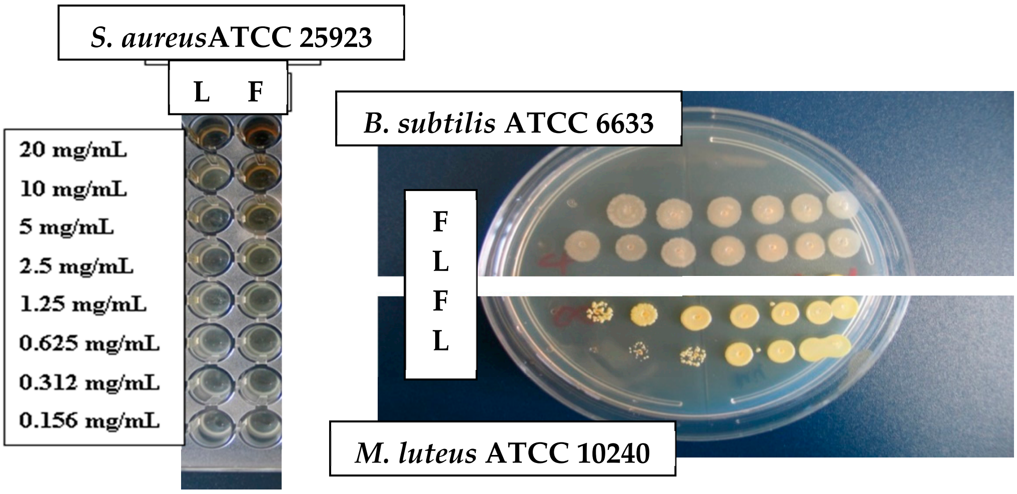

Figure S2. The structures of the metabolites present in the extracts

Alkaloids			
Berberine	Palmatine	Jatrorhizine	N-methylcoclaurine
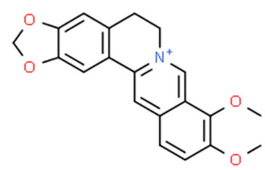	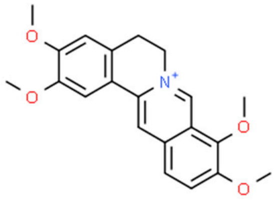	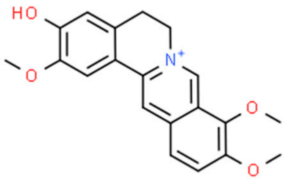	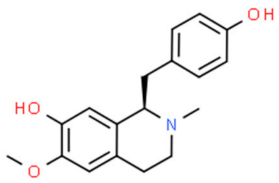
Magnoflorine	Demethyleneberberine	Berberrubine	
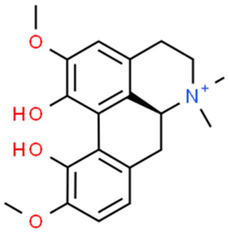	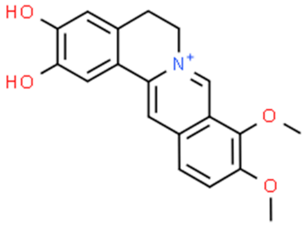	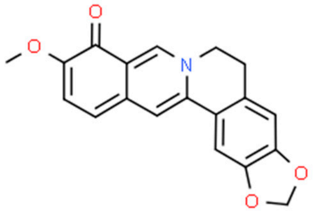	
Berbamunine	Obaberine	Oxyacanthine	Berbamine
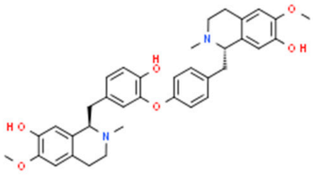	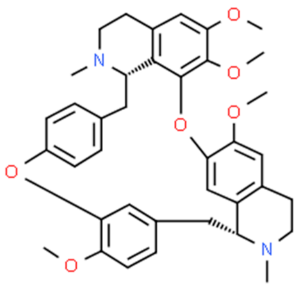	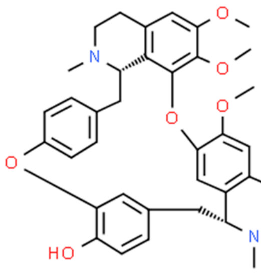	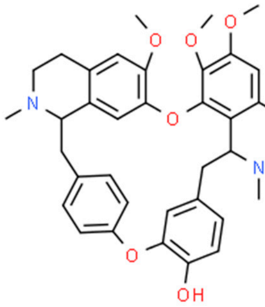
**Phenolic compounds**			
Syringic acid	(Z)-chlorogenic acid	Neochlorogenic acid	Chlorogenic acid
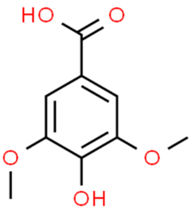	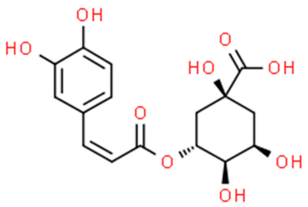	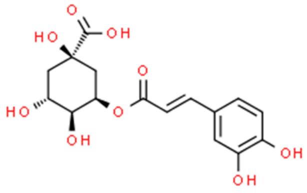	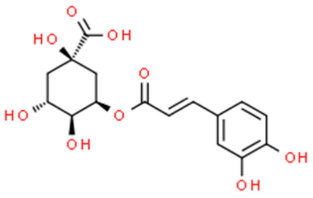
Hydroxybenzoic acid isomers	Caffeic acid	Coumaroyl-quinic acid isomers	Rosmarinic acid

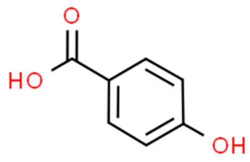
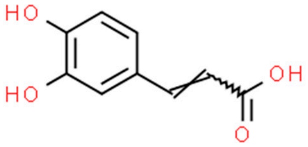
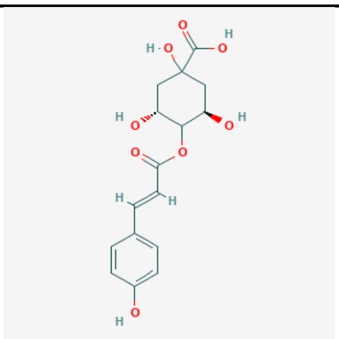
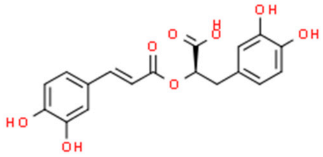

Galloyl-glucose

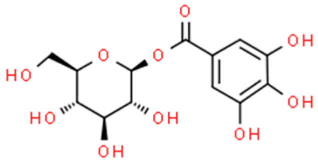

5-Caffeoylglucaric acid

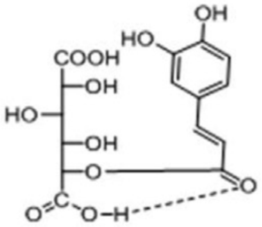

3-Caffeoylglucaric acid

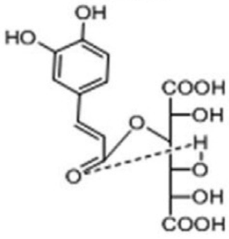

4-Caffeoylglucaric acid

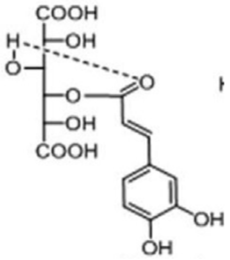

2-Caffeoylglucaric acid

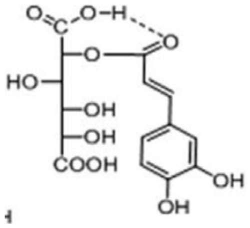

Syringaldehyde

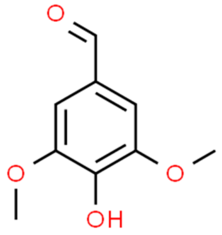

Rutin

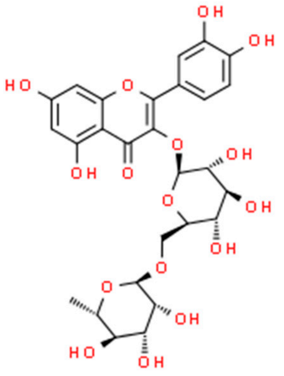

Quercetin

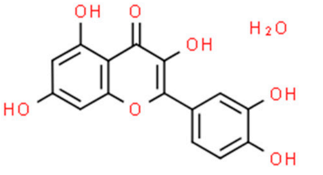

Isoquercetin

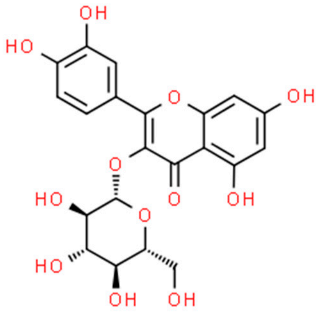

Guaijaverin

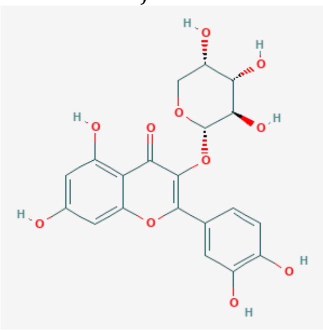

Hesperetin

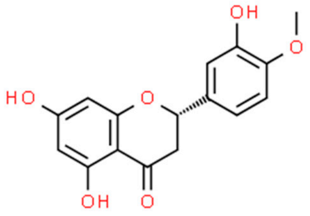

Mandelic acid

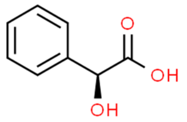

Others

Citric acid

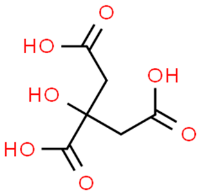

Quinic acid

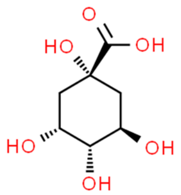

Table S1. Averaged percentage content of phenolic compounds in the studied extracts

	FREtOH	FR70EtOH	FR50EtOH	FRH2O	LFEtOH	LF70EtOH	LF50EtOH	LFH2O	RXEtOH	RX70EtOH	RX50EtOH	RXH2O
**Chlorogenic acid**	0.028	0.023	0.041	0.023	0.012	0.017	0.21	0.15	0	0	0	0
**Neochlorogenic acid**	0.011	0.0089	0.028	0.029	0.0098	0.096	0.086	0.093	0	0	0	0
**Quercetin glucuronide**	0.0018	0.0015	0.0014	0.0018	0.0019	0.0018	0.051	0.071	0	0	0	0
**Isoquercetin**	0.0098	0.0075	0.0069	0.0083	0.021	0.015	0.017	0.028	0.0011	0.0014	0.0045	0.00019
**Kaempferol glucoside**	0.0015	0.0072	0.0081	0.0055	0.0081	0.0092	0.089	0.11	0	0	0	0
**Quercetin**	0.012	0.0077	0.02	0.00088	0.016	0.012	0.015	0.0085	0.00085	0.0012	0.00069	0.00057
**Kaempferol**	0.0086	0.0044	0.011	0	0.022	0.016	0.013	0.0072	0.00099	0.0068	0.0047	0.0024
**3-caffeoylglucaric acid**	0.00067	0.0016	0.0076	0.0087	0.018	0.025	0.1	0.074	0	0	0	0
**4-caffeoylglucaric acid**	0.0015	0.00086	0.0055	0.0054	0.026	0.035	0.096	0.13	0	0	0	0
**2-caffeoylglucaric acid**	0.00012	0.0036	0.0067	0.0063	0.031	0.038	0.14	0.16	0	0	0	0
**5-caffeoylglucaric acid**	0.00061	0.0037	0.004	0.0055	0.014	0.012	0.078	0.082	0.001	0.001	0.0068	0.0082

Table S2. Averaged percentage content of the selected alkaloids in the obtained extracts

	FREtOH	FR70EtOH	FR50EtOH	FRH2O	LFEtOH	LF70EtOH	LF50EtOH	LFH2O	RXEtOH	RX70EtOH	RX50EtOH	RXH2O
**Berberine**	0.4322	0.2656	0.1623	0.1456	0.6719	0.5724	0.5048	0.3746	3.332	2.518	2.1044	1.8018
**Palmatine**	0.0681	0.0573	0.0537	0.0479	0.1843	0.1595	0.1138	0.0811	0.7477	0.6674	0.7095	0.6779
**Jatrorhizine**	0.1779	0.2228	0.174	0.1669	0.7624	0.6365	0.4343	0.3642	1.5985	1.3983	1.3898	1.3625
**Magnoflorine**	0.1356	0.2109	0.223	0.3258	0.6361	0.8575	0.9296	1.3379	0.3088	0.4962	0.5208	0.6455
